# Transient Epileptic Amnesia: A Treatable Cause of Spells Associated With Persistent Cognitive Symptoms

**DOI:** 10.3389/fneur.2019.00939

**Published:** 2019-08-28

**Authors:** Vijay K Ramanan, Kenneth A. Morris, Jonathan Graff-Radford, David T. Jones, David B. Burkholder, Jeffrey W. Britton, Keith A. Josephs, Bradley F. Boeve, Rodolfo Savica

**Affiliations:** Department of Neurology, Mayo Clinic-Rochester, Rochester, MN, United States

**Keywords:** amnestic spells, memory impairment, dementia, neurodegenerative disease, sleep electroencephalogram (EEG)

## Abstract

**Objective:** To characterize the clinical, EEG, and neuroimaging profiles of transient epileptic amnesia (TEA).

**Methods:** We performed a retrospective analysis of patients diagnosed with TEA at the Mayo Clinic Minnesota from January 1, 1998 to September 21, 2017. Diagnostic criteria included the presence of recurrent episodes of transient amnesia with preservation of other cognitive functions and evidence for epilepsy [epileptiform abnormalities on EEG, clinical features of seizures, or symptomatic response to anti-seizure medications (ASMs)].

**Results:** Nineteen patients were identified (14 men, 5 women) with median onset age 66 years and median time to diagnosis 2 years. Thirteen patients (68%) reported persistent cognitive/behavioral symptoms, including 4 (21%) for whom these were the chief presenting complaints. EEG revealed epileptiform abnormalities involving the frontal and/or temporal regions in 12/19 individuals (63%), including activation during sleep in all of these cases. In numerous cases, sleep and prolonged EEG evaluations identified abnormalities not previously seen on shorter or awake-state studies. Brain MRI revealed focal abnormalities in only 4/19 cases (21%). FDG-PET identified focal hypometabolism in 2/8 cases where it was performed, both involving the frontal and/or temporal regions. Anti-seizure therapy, most often with a single agent, resulted in improvement (reduction in spell frequency and/or subjective improvement in interictal cognitive/behavioral complaints) in all 17 cases with available follow-up.

**Conclusions:** TEA is a treatable cause of amnestic spells in older adults. This syndrome is frequently associated with persistent interictal cognitive/behavioral symptoms and thus can be mistaken for common mimics. In the appropriate clinical context, our findings support the use of early prolonged EEG with emphasis on sleep monitoring as a key diagnostic tool. FDG-PET may also complement MRI in distinguishing TEA from neurodegenerative disease when suspected.

## Introduction

Transient epileptic amnesia (TEA) is a clinical presentation of focal epilepsy of presumed temporal origin which is characterized by self-resolving episodes of retrograde and/or anterograde amnesia (1). During episodes, patients may exhibit repetitive questioning and appear confused, disoriented, or anxious, but typically have otherwise preserved neurologic function. Memory for the events themselves can be partially to completely absent, heightening the importance of having witnesses to recount symptoms. Although reminiscent of events of transient global amnesia (TGA), the spells of TEA tend to be shorter (often 1 h or less), more commonly recurrent, associated with waking, and responsive to anti-seizure medications (ASMs) (2, 3).

In addition to the transient amnestic spells which are the hallmark of this disorder, most patients with TEA experience some degree of chronic memory difficulties. Distinctive forms of memory dysfunction associated with TEA include loss over days to weeks of recently learned information (known as accelerated long-term forgetting) and loss of memories of remote life events (known as autobiographical memory impairment) (4, 5). Other less specific cognitive and behavioral complaints can also be present and may precede the identification of amnestic spells by years (6).

Although increasingly recognized as a unique clinical syndrome, TEA can easily be misdiagnosed as TGA (7), migraine variant, or psychogenic amnesia, and may precipitate an evaluation for a neurodegenerative disease due to its typical onset in late adulthood, associated interictal memory complaints, and the possibility for amnestic spells to go unwitnessed or unrecognized (8). Given the therapeutic potential and the strikingly different prognostic implications of this disorder in contrast to those associated with neurodegenerative causes of memory impairment, recognizing TEA is crucial. Our objective was to characterize the clinical, EEG, and neuroimaging profiles of TEA through a detailed review of cases seen at the Mayo Clinic Minnesota.

## Materials and Methods

We performed a retrospective analysis of patients diagnosed with TEA at the Mayo Clinic in Rochester, Minnesota over an ~20-year period between January 1, 1998 and September 21, 2017. Potential cases were identified using the Advanced Cohort Explorer (ACE), a search tool for the Mayo Clinic Enterprise Data Trust. As a broad initial filter for cases where the diagnosis was considered, “transient epileptic amnesia” was queried against the Impression/Report/Plan section of clinical notes.

For the resulting 209 cases, detailed review of the medical record isolated 20 individuals meeting previously proposed diagnostic criteria for TEA (9):

1. recurrent witnessed episodes of transient amnesia
2. cognitive functions other than memory judged to be intact during typical episodes
3. evidence for a diagnosis of epilepsy, based on epileptiform abnormalities (spikes, sharp waves, or temporal intermittent rhythmic delta activity) on EEG, clinical features of seizures (e.g., olfactory hallucinations, premonitory déjà vu, lip-smacking), or a clear-cut symptomatic response (reduction in spell frequency and/or subjective improvement in interictal cognitive/behavioral complaints) to ASMs.

One of these 20 patients was found to have epilepsy related to a pre-existing structural brain injury (intraparenchymal hemorrhage), and was thus excluded from this study to avoid potential confounding, leaving 19 individuals for analysis.

Clinical histories were obtained from the documentation provided by the neurologist at the time of evaluation. Due to the clinical setting and retrospective design of this study, this documentation and the history provided at the encounters was not standardized. A subset of patients (9/19) underwent neuropsychological assessment with test results interpreted by a neuropsychologist. These assessments utilized tools from a standard comprehensive test battery, including evaluations of global cognitive function, premorbid intellectual achievement, learning and memory, attention/concentration, visuospatial functioning, language, and problem solving. The learning and memory tasks typically tested numeric working memory and immediate and 30-min delayed recall of word lists, stories, and visual information utilizing the Wechsler Adult Intelligence Scale-Third Edition (WAIS-III) factors, the Auditory Verbal Learning Test (AVLT), and the Wechsler Memory Scale-Third Edition (WMS-III) Logical Memory and Visual Reproduction subtests. Two of the nine patients with baseline testing had repeat neuropsychological assessment after the initiation of ASM therapy.

A subset of patients underwent neuroimaging. Brain MRI was performed at the time of Mayo Clinic diagnostic evaluation for all 19 patients using 3 Tesla scanners and standard imaging sequences including T1, T2, FLAIR, and DTI. Brain ^18^F-fluorodeoxyglucose positron emission tomography (FDG-PET) scans were obtained at the time of diagnostic evaluation for eight patients, with repeat scan after initiation of ASM therapy for one of these patients. All neuroimaging data was interpreted by a neuroradiologist. For the purposes of this study, generalized atrophy, leukoaraiosis presumed due to small vessel ischemic disease, and chronic lacunar infarcts were not considered major MRI abnormalities. Test results from other institutions were considered as part of the clinical history if raw data was secondarily reviewed by Mayo Clinic physicians.

All study protocols were approved by the local institutional review board.

## Results

Selected clinical characteristics for the 19 patients (14 men, 5 women) included in this study are shown in Table 1. Median age of primary symptom onset was 66 years old, with 16 individuals (84%) noted between 50 and 80 years old at onset. The typical duration of amnestic spells was minutes to a few hours, with a maximum reported duration of 12 h. Transient amnestic events on waking were reported by 5 patients. Average time to diagnosis was 4 years, including 8 individuals (42%) with time to diagnosis exceeding 2 years.

**Table 1:** Demographic and clinical characteristics of individuals included in the study.

**Sex**	**Age^a^**	**Years to Dx**	**CC**	**ABMI^c^**	**ALTF^c^**	**Sleep Sx^d^**	**Psych Sx^d^**	**Migr**	**Other medical history**
M	66	1	Spells	N	N	None	IRR	N	None
M	69	3	COG	Y	N	SNOR; INSM	ANX; IRR	N	Hodgkin's lymphoma
M	54	2	Spells	N	N	None	None	N	Alcohol abuse
M	79	1	Spells	N	N	None	None	N	Meningitis, renal cancer; FH dementia
M	75	2	Spells	N	N	HSMN	DEP	N	None
M	57	1	Spells	N	N	None	None	N	FH TGA
F	59	10	Spells	N	N	None	None	N	None
M	75	0.5	Spells	N	N	None	IRR; DB	N	None
M	62	1	Spells	Y	Y	OSA	None	N	None
M	76	3	Spells	N	N	OSA	ANX	N	None
F	60	2	COG	Y	Y	None	None	Y	TBI
F	68	12	COG^b^	N	Y	None	DEP	N	FH dementia
F	85	1	Spells	N	N	None	None	N	None
M	57	14	BEHAV^b^	N	N	OSA	IRR; DB	N	FH dementia
M	65	5	Spells	N	N	None	None	N	None
F	43	7	Spells	Y	N	OSA	DEP	Y	APS
M	83	6	Spells	N	N	None	LAB	N	None
M	68	2	Spells	N	N	None	None	N	FH epilepsy
M	64	2	Spells	N	Y	None	DEP	N	None

a *Age at onset of primary (chief complaint) symptoms*.

b *Chronic cognitive complaints preceded the identification of amnestic spells*.

c *Symptoms during the syndrome undergoing diagnostic evaluation*.

d *Personal medical history of symptoms (not necessarily isolated to the syndrome undergoing evaluation)*.

*Dx, diagnosis; ABMI, autobiographical memory impairment; ALTF, accelerated long-term forgetting; Y, yes; N, no; CC, chief complaint; Sx, symptoms; COG, cognition; BEHAV, behavioral or personality changes; SNOR, snoring; INSM, unspecified insomnia; HSMN, hypersomnolence; OSA, obstructive sleep apnea; IRR, irritability; DEP, depression; ANX, anxiety; DB, disinhibited behavior; LAB, labile emotions; FH, family history in first degree relative; TGA, transient global amnesia; TBI, traumatic brain injury; APS, antiphospholipid syndrome*.

Amnestic spells represented the most common chief complaint precipitating evaluation (15/19 = 79%). However, 13 patients (68%) reported persistent cognitive and/or behavioral symptoms at the initial evaluation, including 4 (21%) for whom this was the primary complaint and 2 (11%) for whom these symptoms preceded known amnestic spells. Interictally, autobiographical memory impairment and accelerated long-term forgetting were reported by 4 individuals (21%) each. Personal histories of sleep (6/19 = 32%) and psychiatric (10/19 = 53%) symptoms were frequent, though these symptoms were not necessarily concurrent with the presenting complaints. Personal history of migraine headache (2/19 = 11%) and family history of epilepsy (1/19 = 5%) were uncommon. One individual reported a first-degree relative with a TGA event, but no individuals reported family history of TEA.

Office neurologic examination was normal in all patients. Neuropsychological assessment was interpreted as normal or including mild abnormalities in 6/9 cases where it was performed, with only three individuals displaying deficits that would be consistent with mild cognitive impairment (Table 2). In two cases, neuropsychological assessment was repeated at least a year after the initiation of ASM therapy, with at least stability in performance described for both and in one case with improvement in verbal memory (Table 2).

**Table 2:** Neuropsychological assessment and neuroimaging results.

**Sex**	**Age^a^**	**Neuropsych evaluation^b^**	**Brain MRI**	**Brain FDG-PET**
M	66	-	Small encephalocele, L MCF	Normal
M	69	Multidomain amnestic MCI	Normal	-
M	54	-	Normal	-
M	79	-	Foci of restricted diffusion, L hippocampus^c^	-
M	75	Normal	Normal	Normal
M	57	-	Normal	-
F	59	-	Normal	-
M	75	Mildly abnormal (executive dysfunction)	Normal	Hypometabolism, L anteroinferior temporal
M	62	Essentially normal (scattered inefficiencies)	Normal	Normal
M	76	-	Normal	-
F	60	Mildly abnormal (executive dysfunction, word list encoding, visual memory); improved verbal memory and otherwise essentially stable at retest after ASM therapy	Hemosiderin, L temporal	Normal
F	68	Multidomain amnestic MCI	Normal	Normal
F	85	Multidomain non-amnestic MCI	Splenium CC T2 HI	-
M	57	Mildly abnormal (verbal memory, semantic fluency); essentially stable at retest after ASM therapy	Normal	Hypometabolism, L/R frontal, L/R temporal^d^
M	65	-	Normal	-
F	43	Mildly abnormal (isolated test of low memory on paragraph recall, reading inefficiency)	Normal	Normal
M	83	-	Normal	-
M	68	-	Normal	-
M	64	-	Normal	-

a *Age at onset of primary (chief complaint) symptoms*.

b *Performed at time of diagnostic evaluation; additional information is described where testing was repeated following ASM therapy*.

c *Study performed within 24 h of a typical amnestic spell*.

d *Resolved after ASM therapy*.

*MCI, mild cognitive impairment; L, left; R, right; -, not performed; MCF, middle cranial fossa; CC, corpus callosum; HI, hyperintensity*.

**Figure 1:**
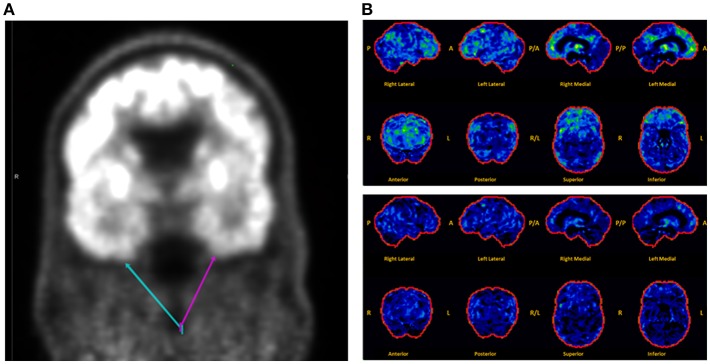
Montage of abnormal brain FDG-PET scans in a case series of transient epileptic amnesia. Representative images are displayed for two individuals meeting diagnostic criteria for TEA who displayed focal hypometabolism on FDG-PET. **(A)** This 75-year-old man was evaluated for 6 months of amnestic spells, irritability, and disinhibition, and displayed subtle focal hypometabolism in the anteroinferior aspect of the left temporal lobe (magenta arrow) as compared to the right (teal arrow). **(B)** This 71-year-old man presented for evaluation of longstanding personality changes (starting at age 57) and more recent recurrent spells of amnesia and confusion, and displayed normal brain MRI and left frontal and right frontotemporal epileptiform abnormalities on EEG during sleep. (Top Panel) Brain FDG-PET prior to initiation of antiepileptic therapy showed hypometabolism (indicated by regions in green and yellow) most marked in the left medial frontal, anterior cingulate, and medial temporal regions. (Bottom Panel) Brain FDG-PET 1 year after initiation of lamotrigine showed extensive resolution of the previously visualized hypometabolism. The patient clinically improved during that time, with resolution of both the personality changes and spells. A, anterior; P, posterior; R, right; L, left.

Brain MRI revealed focal abnormalities in only 4 cases (Table 2). Brain FDG-PET was interpreted as normal in 6/8 cases where it was performed (Table 2). Focal hypometabolism was identified on FDG-PET in two cases, both involving the frontal and/or temporal regions (Figure 1) and both preceded by normal brain MRIs. One of those cases involved a 71-year-old man who 14 years prior (at age 57) had developed irritability, disinhibition, and mild forgetfulness, with evaluations at that time suggesting the possibility of an emerging behavioral variant frontotemporal dementia due to his symptoms, mild abnormalities of executive function on neuropsychological assessment, and mild hypoperfusion in the frontal and temporal lobes on SPECT scan. Years later, TEA was diagnosed after recurrent amnestic spells were observed by family and an EEG (preceded by a normal study 8 months prior) revealed frontal and temporal epileptiform abnormalities in sleep. Lamotrigine therapy resulted in cessation of his amnestic spells, resolution of his behavioral symptoms, and subjectively reported improvement in his recent memory. FDG-PET prior to lamotrigine therapy revealed hypometabolism of the left frontal and temporal regions which had resolved on repeat scan 1 year after ASM initiation (Figure 1B).

Epilepsy-associated clinical features such as olfactory hallucinations or repetitive chewing were associated with amnestic spells in 7 patients (37%) (Table 3). EEG revealed epileptiform abnormalities involving the frontal and/or temporal regions in 12 individuals (63%), with a preponderance of these cases (10/12 = 83%) including bilateral abnormalities. Ten patients in the series underwent prolonged EEG monitoring (≥24 h), with all of these recordings displaying epileptiform abnormalities, including in 4 cases where a previous routine EEG (<1 h) was normal. Sleep activation of EEG abnormalities was identified in all cases with epileptiform abnormalities, including 5 individuals who displayed new epileptiform abnormalities in sleep, 4 individuals who displayed epileptiform abnormalities only in sleep, and 1 individual who had electrographic seizures during sleep which were not observed during wakefulness. Overall, 13 patients (68%) demonstrated an abnormality of some kind (EEG, MRI, or FDG-PET) involving the frontal and/or temporal regions.

**Table 3:** EEG results and associated clinical epilepsy-related features.

**Sex**	**Age^a^**	**Amnestic spell duration/frequency**	**Epilepsy features**	**EEG epileptiform abnormalities**	**Bilateral epileptiform**	**Normal routine EEG before prolonged**	**ASMs failed**	**ASMs successful**	**EEG sleep activation**	**Seizure during sleep**	**EEG activation only in sleep**	**New EEG activation in sleep**
M	66	8 over 1 year/several hours	None	L/R temporal^b^^,^ ^c^	Y	Y	–	OXC	Y	N	N	N
M	69	9 over 1 year/ <1 h	None	L/R frontotemporal^b^	Y	N	LTG	CBZ	Y	N	N	Y
M	54	10 over 1 year/few hours	Premonitory electrical sensation	None	N	n/a		LTG	N	N	N	N
M	79	4–6 over 1 year/1–12 h	None	None	N	n/a		LEV	N	N	N	N
M	75	3 over 1 year/1 h	Olfactory hallucinations	None	N	n/a		LTG	N	N	N	N
M	57	4 over 6 months/ <1 h	Olfactory hallucinations	None	N	n/a			N	N	N	N
F	59	5 over 1 year/ <30 min	None	L temporal^b^	N	Y			Y	N	N	N
M	75	3 over 4 months/ <1 h	None	L temporal^b^^,^ ^c^	N	N		LTG	Y	N	N	N
M	62	6 over several months/unknown	Olfactory hallucinations	L/R temporal^b^	Y	N		OXC	Y	N	Y	N
M	76	3 per year/ <20 min	Postictal drowsiness	L/R temporal^b, c^	Y	Y		LEV	Y	N	Y	N
F	60	3 over 2 years/several hours	None	L/R frontotemporal	Y	N		LTG; LEV	Y	N	N	Y
F	68	0–3 per week/ <20 min	None	L/R temporal	Y	N	LTG	LEV	Y	N	N	N
F	85	3 over 1 year/several hours	None	L/R temporal	Y	n/a		LEV	Y	N	N (also with HV)	N
M	57	1–2 per month/several hours	None	L/R frontotemporal^c^	Y	n/a		LTG	Y	N	Y	Y
M	65	<1 per year/several hours	None	None	N	n/a		LEV	N	N	N	N
F	43	2 per month/minutes to 1–2 h	None	L/R frontotemporal^b, c^	Y	Y		LEV; OXC	Y	Y	Y	
M	83	6–7 over 18 months/several h	Olfactory hallucinations	None	N	n/a	LEV^d^; LTG^d^	LAC	N	N	N	N
M	68	4 over 1 year/10–30 min	Known other seizure events	None	N	n/a		OXC	N	N	N	N
M	64	Unknown/minutes to several hours	None	L/R temporal	Y	N		CBZ	Y	N	N	Y

a *Age at onset of primary (chief complaint) symptoms*.

b *Findings from prolonged (at least 24 h of monitoring) EEG*.

c *Exhibited at least one normal short-term EEG during their workup prior to diagnosis*.

d *Clinical response (spell reduction or memory improvement) but did not tolerate due to side effects*.

*Y, Yes; N, No; LTG, lamotrigine; LEV, levetiracetam; OXC, oxcarbazepine; CBZ, carbamazepine; ZON, zonisamide; LAC, lacosamide; n/a, not applicable due to prolonged EEG not being performed; HV, hyperventilation*.

All 19 patients in the series were prescribed ASMs, with 17 (89%) having documented clinical follow-up after ASM initiation and 2 (11%) patients lost to follow-up. Among individuals with available follow-up data, all exhibited clinical improvement (defined as a reduction in spell frequency and/or improvement in self-reported interictal cognitive/behavioral complaints) with treatment, in almost all cases (15/17 = 88%) with a single agent (Table 3). Levetiracetam (7/17 = 41%), carbamazepine/oxcarbazepine (6/17 = 35%), and lamotrigine (5/17 = 29%) were the most commonly successfully utilized drugs.

## Discussion

Our study reinforces that TEA is a potentially treatable cause of amnestic spells which can be associated with persistent interictal cognitive and behavioral symptoms in older adults. Our results based on retrospectively acquired data provide independent confirmation of core syndromic features of TEA which were previously characterized through a prospective design at another center (1). The EEG findings from this series are also consistent with the understanding of TEA as a manifestation of focal seizures of temporal lobe origin (1, 6, 10, 11), and support the value of prolonged EEG with emphasis on sleep monitoring in suspected patients when routine EEG is unremarkable. In addition, our data suggest that FDG-PET may complement MRI with its ability to rule out neurodegenerative disease when suspected, potentially identify other patterns of hypometabolism suggestive of TEA, and monitor for metabolic response to ASM therapy.

**Figure 2:**
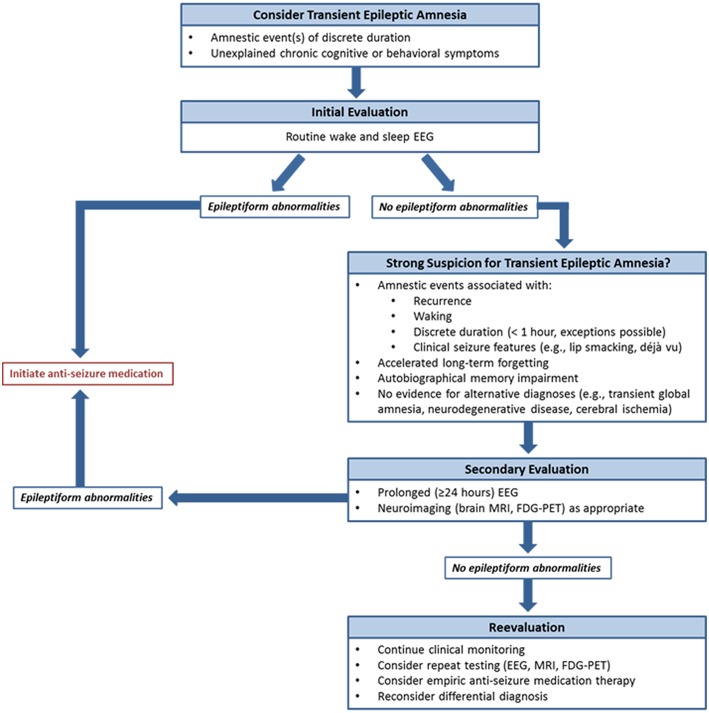
Proposed diagnostic algorithm for transient epileptic amnesia. To assist clinicians in the evaluation of suspected TEA, a diagnostic algorithm is proposed.

EEG was a critical diagnostic element in this series. As in previous reports (4–6), epileptiform abnormalities arose from the frontotemporal regions, most often present independently from both temporal regions. These patients often had no corresponding structural abnormality on brain MRI. In several cases, prolonged EEG monitoring identified abnormalities not seen on prior short-term studies, likely due to both the presence of longer recording and the extended capture of sleep with its proclivity for activation of epileptiform activity (12). These observations parallel findings from the largest TEA series to date, where nearly half of individuals displayed interictal epileptiform abnormalities exclusively during sleep (1). Sleep is known to be crucial for memory consolidation (13), and frequent nocturnal subclinical seizures have been proposed as mechanisms for unremitting accelerated long-term forgetting and autobiographical memory impairment in TEA (4, 11). In addition, comorbid sleep pathologies such as obstructive sleep apnea can impact seizure frequency (14). These concepts suggest that prolonged EEG including sufficient sleep recording may be crucial both for initial diagnosis, particularly when short-term studies are unrevealing, and for assessing for uncontrolled subclinical seizures if interictal cognitive and behavioral symptoms do not improve despite ASM initiation. Given the frequent delays to diagnosis observed in this series, and the expected negative effects of this on quality of life and health care expenditures among other factors, our findings argue for a low threshold to proceed to prolonged EEG monitoring when there is clinical suspicion for TEA and routine EEG is normal. Another relevant point of our results is also the relative delay in diagnosis of TEA; in our series we observed cases with up to 14 years of symptoms before diagnosis. We strongly recommend considering the possibility of TEA in patients with amnestic syndromes of discrete duration, which may lead to earlier initiation of therapy of a treatable condition (Figure 2).

Prior neuroimaging studies of TEA have described subtle regional correlates for its associated memory deficits, including atrophy of the hippocampi on MRI (15, 16) and hypometabolism of medial temporal structures on FDG-PET (6) as associated with autobiographical memory impairment. Although the basis for accelerated long-term forgetting, autobiographical memory impairment, and non-specific memory complaints in TEA is not yet known, disruption of neural networks for memory consolidation has been proposed as a putative mechanism (17, 18).

In this series, brain MRI was normal or included only minor/incidental abnormalities in almost all cases, and FDG-PET was clinically interpreted as normal in most cases where it was performed. In the few cases where FDG-PET included abnormalities, these were limited to focal hypometabolism involving the frontal and/or temporal regions and did not fit known patterns suggestive of an underlying neurodegenerative disease. In addition, we identified a case with widespread cortical hypometabolism which resolved following ASM therapy and resulting clinical improvement. This concept of dynamic changes in brain function over the history of the disorder in part parallels a previous report of left hippocampal hypermetabolism on FDG-PET during an amnestic spell, with subsequent resolution 1 month later (19). FDG-PET may also be a useful complement to brain MRI in cases where TEA is suspected but the initial workup is unrevealing or where evaluating for neurodegenerative disease is a consideration. In particular, although our results suggest that patients with TEA can exhibit frontal and/or temporal hypometabolism, the potential for reversibility of these imaging findings with ASM therapy may be an additional discriminator vs. a frontotemporal dementia (when the latter is a consideration in the differential diagnosis). Further study is needed to identify whether a common signature pattern of hypometabolism (rather than simply the absence of a neurodegenerative pattern) exists on FDG-PET for TEA.

Most patients in this series clinically responded to a single ASM, with levetiracetam and lamotrigine as the most commonly utilized medications. This clinical response frequently included improvement in both spell frequency and interictal cognitive and behavioral symptoms, with the caveat that interictal symptoms were most often subjectively reported and mild even prior to treatment. Although our study was not designed to compare efficacy among ASMs, in this series medications were typically switched due to side effects rather than a lack of clinical response, suggesting that TEA is generally responsive to ASM therapy.

For this study, clinical improvement was defined as reduction in the frequency of amnestic spells and/or improvement in subjectively reported cognitive or behavioral complaints. In our TEA series as in others, at diagnostic evaluation most patients exhibited normal or essentially normal (i.e., only mildly abnormal) neuropsychological assessment utilizing standard test batteries, which typically do not include specific objective measures of autobiographical memory impairment or accelerated long-term forgetting (4, 9). In addition, the number of patients in our series who underwent repeat neuropsychological assessment after initiation of ASM therapy was prohibitively small to be able to assess for quantitative improvement in cognition. Within these limitations as well as the possibility for effects of certain ASMs (such as lamotrigine) to positively impact mood, we are not able to directly conclude based on this study that patients with TEA may experience objective improvement in cognition following ASM therapy. Nevertheless, the improvement in subjective cognitive symptoms (which were often corroborated by informants) does in our view constitute a meaningful clinical improvement, particularly in view of the potential for uncontrolled seizures to cause cognitive symptoms (20) and the evolving literature on subjective cognitive decline as a potential preclinical marker of impending objective change (21, 22).

Our study has a number of limitations. As the case series was identified through retrospective chart review, not all individuals included had all possible diagnostic data or a uniform approach to testing. In addition, though previous studies of TEA suggested an overall benign course without increased risk of dementia (10), our case series had high variability in the extent of follow-up, limiting the ability to draw conclusions about long-term prognosis. Key clinical features such as accelerated long-term forgetting, autobiographical memory impairment, and waking-predominance of spells, may not have been systematically assessed during patient interviews and thus their frequencies here may not accurately reflect their prevalence in the general population with TEA. Our series predominantly included older adults, and further study is needed to determine whether there is a biological rationale for TEA to present at a late age vs. whether it may be relatively underrecognized in younger individuals. Finally, in most cases in this series, clinical improvement was judged based on history (subjective report of resolution of spells and/or cognitive/behavioral symptoms), highlighting the potential value of a novel neuroimaging, electrographic, or neuropsychometric biomarker to monitor for response to treatment.

The inclusion criteria for this study were based on previous enumerations about TEA (9). Through our initial review, we also encountered cases where a TEA phenotype was suspected due to the presence of archetypal memory complaints (accelerated long-term forgetting or autobiographical memory impairment) and evidence for epilepsy (on EEG, by history, or via clinical improvement with ASM therapy) despite the absence of recurrent witnessed amnestic spells. Although these cases did not meet criteria for inclusion in this study, given the possibility for ictal events to be missed (for being unwitnessed, unrecognized, or unregistered) or subsequent to preceding interictal cognitive or behavioral symptoms, these observations suggest a potential need for widening of the conceptual phenotypic spectrum of TEA, particularly in clinical settings where empiric anti-seizure therapy can provide potentially useful diagnostic information. These observations are similar in concept to the proposed entity of epileptic amnesic syndrome, defined by the presence of senile-onset persistent memory complaints in association with often unrecognized (due to being brief, non-disabling, and possibly unwitnessed) seizures (23). They also mirror the description of a case where autobiographical memory impairment and accelerated forgetting preceded by several years the presentation of transient spells of cognitive disturbance confirmed by a witness and ictal epileptic activity on EEG as TEA (24). Given its typically positive response to treatment and otherwise benign long-term prognosis, accurate diagnosis of TEA is crucial to facilitate early intervention to prevent morbidity due to uncontrolled amnestic seizures and cognitive symptoms.

## Conclusion

TEA is a treatable cause of amnestic seizures in older adults which is frequently associated with interictal cognitive/behavioral complaints in older adults and often has a significant lag time to diagnosis. In the appropriate clinical context, our findings support the use of EEG (particularly with prolonged studies including sleep) and FDG-PET to help confirm the diagnosis against common mimics and to serve as potential future biomarkers.

## Data Availability

All datasets generated for this study are included in the manuscript.

## Author Contributions

VR and RS were involved in initial study design and data analyses as well as drafting of the manuscript. All authors reviewed and edited the manuscript and provided critical intellectual content during execution of the project.

### Conflict of Interest Statement

JG-R and KJ receive funding from the National Institutes of Health. DJ receives funding from the National Institutes of Health and the Minnesota Partnership for Biotechnology and Genomics. JB is an unpaid co-investigator for the following studies: a double-blind, randomized, placebo-controlled study to investigate the efficacy and safety of cannabidiol (GWP42003-P, CBD) as add-on therapy in patients with tuberous sclerosis complex who experience inadequately-controlled seizures; a double-blind, randomized, placebo-controlled study of IVIG in patients with voltage-gated potassium channel complex antibody-associated autoimmune epilepsy; an open label extension study to investigate the safety of cannabidiol (GWP42003-P; CBD) in children and adults with inadequately controlled Dravet or Lennox-Gastaut syndromes; and a study on the compassionate use of stiripentol in an intractable epilepsy due to Dravet syndrome and malignant migrating focal epilepsy of infancy. BB has served as an investigator for clinical trials sponsored by Axovant and Biogen; he receives royalties from the publication of a book entitled Behavioral Neurology of Dementia (Cambridge Medicine, 2009, 2017); he serves on the Scientific Advisory Board of the Tau Consortium; and he receives research support from the NIH, the Mayo Clinic Dorothy and Harry T. Mangurian Jr. Lewy Body Dementia Program, and the Little Family Foundation. The remaining authors declare that the research was conducted in the absence of any commercial or financial relationships that could be construed as a potential conflict of interest.
